# Parents and adolescents preferences for asthma control: a best-worst scaling choice experiment using an orthogonal main effects design

**DOI:** 10.1186/s12890-015-0141-9

**Published:** 2015-11-17

**Authors:** Wendy J. Ungar, Anahita Hadioonzadeh, Mehdi Najafzadeh, Nicole W. Tsao, Sharon Dell, Larry D. Lynd

**Affiliations:** Program of Child Health Evaluative Sciences, The Hospital for Sick Children, Peter Gilgan Centre for Research and Learning, 11th floor, 686 Bay Street, Toronto, Ontario M5G 0A4 Canada; The Institute for Health Policy, Management & Evaluation, University of Toronto, Toronto, Ontario Canada; Division of Pharmacoepidemiology, Department of Medicine Harvard Medical School, Boston, MA USA; Faculty of Pharmaceutical Sciences, University of British Columbia, Vancouver, BC Canada; Department of Pediatrics, University of Toronto, Toronto, Ontario Canada; Centre for Health Evaluation and Outcome Sciences, Providence Health Research Institute, Vancouver, BC Canada

**Keywords:** Adolescents, Asthma control, Best-worst scaling, Child health, Preferences

## Abstract

**Background:**

The preferences of parents and children with asthma influence their ability to manage a child’s asthma and achieve good control. Potential differences between parents and adolescents with respect to specific parameters of asthma control are not considered in clinical asthma guidelines. The objective was to measure and compare the preferences of parents and adolescents with asthma with regard to asthma control parameters using best worst scaling (BWS).

**Methods:**

Fifty-two parents of children with asthma and 44 adolescents with asthma participated in a BWS study to quantify preferences regarding night-time symptoms, wheezing/chest tightening, changes in asthma medications, emergency visits and physical activity limitations. Conditional logit regression was used to determine each group’s utility for each level of each asthma control parameter.

**Results:**

Parents displayed the strongest positive preference for the absence of night-time symptoms (β = 2.09, *p* < 0.00001) and the strongest negative preference for 10 emergency room visits per year (β = −2.15, *p* < 0.00001). Adolescents displayed the strongest positive preference for the absence of physical activity limitations (β = 2.17, *p* < 0.00001) and the strongest negative preference for ten physical activity limitations per month (β = −1.97). Both groups were least concerned with changes to medications.

**Conclusion:**

Parents and adolescents placed different weights on the importance of asthma control parameters and each group displayed unique preferences. Understanding the relative importance placed on each parameter by parents and adolescents is essential for designing effective patient-focused disease management plans.

**Electronic supplementary material:**

The online version of this article (doi:10.1186/s12890-015-0141-9) contains supplementary material, which is available to authorized users.

## Background

Despite the availability of asthma guidelines [1–3], achieving control continues to be challenging for children, parents and health providers [4–8]. Research has shown that adult asthma patients [9, 10] and parents [11, 12] have unique perceptions of asthma control. Since children depend on parents/caregivers for access to health services and use of medications, parental preferences play an important role in asthma control. The Global Initiative for Asthma (GINA) recommends consideration of patient preferences for adults and for children over 5 years as they relate to treatment choice, peak flow meter use, cost and other issues related to treatment [2]. The US Expert Panel Report (EPR) 3 guidelines recommend consideration of preferences, concerns and school schedules in selection of treatments for children aged 5 to 11 years and for children aged 12 years and older [1]. None of the guidelines considers preferences with regard to actual parameters of asthma control, which may affect adherence to disease management plans.

Best-worst scaling (BWS) experiments offer a rigourous method for measuring preferences [13, 14]. In a BWS study, respondents choose the most and the least preferred items from a list of three or more attributes presented in a single task. A questionnaire typically contains ten to twenty choice tasks with the values of the attributes changing for each choice task. By analysing the respondents’ selections over a series of choice tasks, the preferences for each attribute level can be estimated statistically [15]. BWS questionnaires are easy to administer, making this approach ideal for child health research [16]. The study objectives were to measure and compare the preferences of parents of young children with asthma and adolescents with asthma regarding attributes of asthma control.

## Methods

### Item selection

Potential attributes and levels related to asthma control were initially identified through a literature review. Attributes were discussed in two parent focus groups and one adolescent focus group. Parents and teens were not related to each other. Focus group participants presented their views on factors they perceived to be important in asthma control. Focus groups were audiotaped and transcripts were coded for recurrent themes. Factors important for asthma control clustered around symptoms, medication changes, school and work absences, physical activity limitations, emergency services, season and susceptibility to colds. A draft BWS instrument with five asthma control attributes was created for pilot testing [1–3, 17].

### BWS instrument development

As a full factorial design which would encompass all possible combinations of attribute levels would not be feasible, a fractional design that included a plausible array of choice options was created using Sawtooth™ software (Sequim, WA). A subset of these choice tasks was selected to adhere to BWS design principles to maximize design efficiency. This included displaying i) orthogonality which ensures that differences in the levels of each attribute vared independently over choice sets, and ii) balance, to confirm that all levels appeared with equal frequency in the questionnaire [15]. Using an orthogonal main effects plan design, a questionnaire with 18 choice tasks was created. To avert respondent fatigue, this questionnaire was divided into two blocks or versions, each with nine choice tasks. Each task contained the same five attributes, with levels that varied across tasks. Each task asked respondents to choose the most and least preferred items (Table 1, Additional file 1). The questionnaire took 10 to 20 minutes to complete.

**Table 1:** A sample best-worst choice task

Considering the following choices of attributes and their levels, please indicate which one you consider as the most preferred (best) and which one you consider as the least preferred (worst) attribute in asthma control. Please choose only one best and only one worst.		
Best		Worst
□	Night-time symptoms:	□
	None	
□	Wheezing or tightening of chest:	□
	Chest tightening or wheezing, but it is manageable (does not worsen)	
□	Changing medication:	□
	More doses or adding on another medication needed	
□	Emergency visits:	□
	4 Emergency room visits per year	
□	Limitation of physical activities:	□
	10 limitations per month	

The draft instrument was pilot-tested in 18 English-speaking parents of children with clinically diagnosed asthma and unrelated adolescents with asthma. The pilot test resulted in minor revisions. The final instrument contained 5 attributes with 3 levels each. The attributes and levels were: Night-time symptoms (none; 3 days per week; 5 days per week), Wheezing or tightening of chest (no chest tightening or wheezing; chest tightening or wheezing but it is manageable [does not worsen]; chest tightening or wheezing and is bothersome [may worsen]); Changing medication (no changes needed to medication; more doses or adding on another asthma medication needed; adding oral steroids for 5 days needed), Emergency visits (no Emergency room visits; 4 Emergency room visits per year; 10 Emergency room visits per year) and Participation in physical activities (no physical activity limitations; 2 limitations per month; 10 limitations per month). Numeric values for levels were designated rather than ordinal values (e.g. never, occasionally and often) so that respondents would interpret each level in the same way. The use of extreme values (e.g. Night-time symptoms 5 days per week, Emergency room visits 10 per year) is useful in BWS experiments to enable measurement of negative as well as positive preferences. A reasonable time interval was selected for each attribute (e.g. week, month or year). For example, an interval of one month was selected for activity limitations as children typically participate in organized physical activity on a weekly basis over several months and parents and teens could therefore express their preferences for the indicated levels without difficulty. Similarly, parents have been shown to be reliable reporters of asthma emergency room visits over one year [18] and choosing this interval allowed for the specification of a wide frequency range.

### Participants

The target populations were parents with a child with clinically diagnosed asthma between 2 and 12 years of age or adolescents with asthma aged 12 to 16 years who had a prescription for an asthma controller in the previous year. Children with significant respiratory, cardiac, neurological and congenital conditions, cancer, musculoskeletal abnormalities and psychological or mental health impairments were excluded. Respondent groups were unrelated and the same family could participate in only one of the two surveys. Focus group and pilot study participants were excluded. Parent and adolescent respondents were recruited from two outpatient asthma clinics, an asthma education clinic and through the Asthma Society of Canada (ASC), a community advocacy organization.

### Data collection

Potential subjects were randomly allocated to one of two BWS questionnaire blocks. Packages containing an information sheet, the BWS instrument, a parent-completed demographics questionnaire, and a parent-completed child health questionnaire were mailed to interested participants. The demographics questionnaire captured child age, child sex, parental education, whether the reporting parent was born in Canada, whether the child had access to a drug plan and household income. These items were selected to permit a characterization of the socioeconomic status of the sample and facilitate comparison with other studies. As colds were identified as an important factor for asthma worsening during the focus groups, questions related to colds were included in the health questionnaire. These questions used a 6-month recall interval shown to be reliable in previous studies [18]. For adolescents, their parents completed the health and demographics questionnaires only. The questionnaires could be completed by mail, phone or online.

### Statistical analysis

Data from parent and adolescent samples were analysed separately. Demographic and health data were analysed with descriptive statistics using SAS version 9.2 (Carey, NC). Conditional logit regression with choice as the dependent variable was used to analyse the BWS data and generate regression coefficients for attribute levels using Latent Gold Choice, version 4.5 (Belmont, MA). A main effects model in which the regression coefficients represent the strength of preference for the specified level for each group was constructed. This analysis allows a comparison of preferences for all levels of all attributes relative to a single reference level. Ten physical activity limitations per month was designated as the reference level because its utility fell between more preferred attribute levels (e.g. no night-time symptoms) and less preferred levels (e.g. ten emergency visits). A standard error, p value and 95 % confidence intervals (CI) were calculated for each coefficient based on all choices within each group, without adjustment for clustering of preferences at the respondent level. The main effects analysis did not assess statistical interactions between attribute levels and sociodemographic variables.

### Ethics, consent and permissions

The study was approved by the Research Ethics Boards of The Hospital for Sick Children (Sick Kids), the University of British Columbia-Providence Health Care Research Institute, and the William Osler Health System, Canada (adolescent study only). Completion of the questionnaires signified informed consent. Participants were not asked to consent to making their data public.

## Results

Of 80 packages mailed, 50 parents (63 %) completed health and demographic questionnaires and 52 (65 %) completed the choice questionnaire. Of 88 packages mailed to adolescents, 54 (62 %) parents of adolescents completed health and demographic questionnaires and 44 adolescents (50 %) completed the choice questionnaire.

### Sample characteristics

Parents in both groups were comparable on most characteristics (Table 2). A higher proportion of parent group respondents received a university education compared to parents of adolescents. There was a trend toward greater respiratory health services use in the parent group. Ninety percent of the children of the parent sample were using a controller with or without other medications compared to 85 % in the adolescent sample. Twenty-six percent of the children of parent respondents used combination long-acting beta agonist plus inhaled corticosteroid medications compared to 59 % of adolescents, however the former group represents younger children for whom combination inhalers may not be indicated. The sample displayed a symptom frequency, rate of exacerbations and use of urgent care indicative of moderate to severe asthma for the children of parent participants as well as the adolescents.

**Table 2:** Demographic and health characteristics

Characteristic	Parents (*n* = 50)		Adolescents (*n* = 54)		*P*-value
	n	%	n	%	
Child’s age (years), mean (SD)	7.6 (2.5)		13.5 (1.2)		**<0.0001**
Child of male sex	32	64.0	36	66.7	0.84
Parents born in Canada	27	54.0	31	57.4	0.84
Parental education					**0.0255**
University or college degree/diploma	38	76.0	31	57.4	
Some university or college	7	14.0	8	14.8	
Completed high school or less	5	10.0	15	27.8	
Family has a drug benefits plan	41	82.0	42	77.8	0.59
Annual household income ($ CDN)					0.43
Less than $60,000	11	22.0	19	35.2	
$60,000 to $120,000	17	34.0	16	29.6	
Greater than $120,000	15	30.0	6	11.1	
Not sure or prefer not to respond	7	14.0	13	24.1	
Asthma attacks in last 6 months, mean (SD)	2.6 (3.7)		1.8 (3.6)		0.29
History of other respiratory conditions					
Respiratory syncytial virus	8	16.0	1	1.9	**0.0134**
Croup	15	30.0	5	9.3	**0.0116**
Symptom frequency in the last month					0.99
None	13	26.0	16	29.6	
One to two times per month	14	28.0	15	27.8	
One to three times per week	14	28.0	15	27.8	
One to four times per day	7	14.0	7	13.0	
Other or missing	2	4.0	1	1.9	
Night-time asthma symptoms in last month	31	62.0	26	48.1	0.20
≥1 family doctor visit in last 6 months	18	36.0	11	20.4	0.19
≥1 pediatrician visit in last 6 months	13	26.0	9	16.7	0.55
≥1 respiratory specialist visit in last 6 months	35	70.0	36	66.7	0.39
≥1 emergency room visit in last year	13	26.0	16	29.6	0.21
≥1 hospital admission in last year	3	6.0	7	13.0	0.18
Received asthma management or action plan	35	70.0	40	74.1	0.79
Exposed to second-hand smoke in public	6	12.0	10	18.5	0.42
Asthma medications used in last year					0.35
BD monotherapy	2	4.0	4	7.4	
ICS monotherapy	4	8.0	1	1.9	
BD + ICS or BD + AL	25	50.0	33	61.1	
BD + ICS + AL	16	32.0	12	22.2	
Oral corticosteroid with or without other asthma medication	2	4.0	4	7.4	
Other	1	2.0	0	0.0	

*Abbreviations*: *AL* anti-leukotriene, *BD* bronchodilator, *ICS* inhaled corticosteroid, *SD* standard deviation. *P* value from t-test for continuous variables and Fishers Exact test for categorical variables. Significant results indicated in bold type

Parents’ reports of physical activity limitations and the effects of colds on their child’s asthma were similar for young children and for adolescents (Table 3). Eighty percent of parents of young children and 76 % of parents of adolescents with asthma reported their children being equally or more physically active than other children of the same age, but just over half reported that their children catch colds, flu or respiratory infections more easily. A majority of both groups reported worsening of asthma due to colds.

**Table 3:** Physical activity and cold symptoms

Characteristic	Parents (*n* = 50)		Adolescents (*n* = 54)		*P*-value
	n	%	n	%	
Parent report of child’s physical activity compared with other children of same age					0.25
Much more active	13	26.0	17	31.5	
Moderately more active	9	18.0	10	18.5	
Equally active	18	36.0	14	25.9	
Moderately less active	7	14.0	13	24.1	
Not sure	3	6.0	0	0.0	
Child catches colds, flu or respiratory infections more easily than other children	29	58.0	29	53.7	0.70
Frequency of colds in last 6 months					0.88
Never	4	8.0	5	9.3	
1 to 2 times	23	46.0	28	51.9	
3 to 4 times	14	28.0	14	25.9	
More than 4 times	9	18.0	7	13.0	
Number of days cold lasts					
3 to 5 days	19	38.0	28	51.9	0.49
6 to 7 days	15	30.0	11	20.4	
More than a week	12	24.0	10	18.5	
Not applicable	4	8.0	5	9.3	
Worsening of asthma due to colds	41	82.0	40	74.1	0.67
Frequency of asthma worsening due to colds in last 6 months					0.41
Never	14	28.0	18	33.3	
About half of the times child has a cold	13	26.0	14	25.9	
Every time child has a cold	20	40.0	22	40.7	
Other	3	6.0	0	0.0	

*P* value from Fishers Exact test

### Preferences for asthma control

**Fig. 1:**
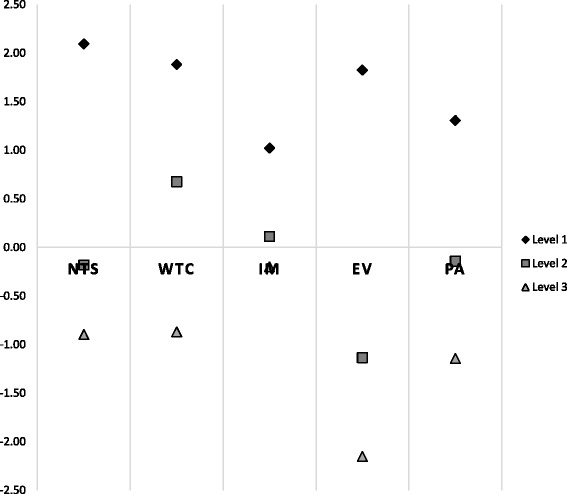
Parent preferences for asthma control. The figure indicates the strength of preference of parents for each of the levels of each asthma control parameter, with positive values indicating more favoured options. Abbreviations: EV, Emergency room visits; IM, Changing medication; NTS, Night-time symptoms; PA, Physical activity limitations; WTC, Wheezing or tightening of chest

The regression analysis of parents’ preferences are presented in Table 4 and plotted in Fig. 1. The results indicate that parents had the strongest positive preference for the absence of night-time symptoms (β = 2.09, *p* < 0.00001), which was greater than their preference for the absence of wheezing or chest tightening, no changes to medications, no emergency room visits and no physical activity limitations. Parents’ strongest negative preference (least favourable) was for 10 emergency room visits per year (β = −2.15, *p* < 0.00001) which was much less preferred than frequent night-time symptoms, bothersome wheezing or chest tightening, the need to add oral steroid medications or ten physical activity limitations per month. The spread between the most and least preferred levels for each attribute is an indication of the relative importance of that attribute to respondents, given the level options specified in the BWS questionnaire. The wide magnitude for emergency room visits suggests that parents strongly distinguished between zero and ten emergency room visits per year. Their preferences between the highest and lowest levels of night-time symptoms, wheezing or chest tightening and physical activity limitations were similar. Parents expressed the least difference in their preferences with regard to the need to make changes to their child’s asthma medications. While parents expressed a negative preference for the addition of an oral corticosteroid (β = −0.20), they were only slightly more averse to this than to night-time symptoms three days per week (β = −0.18) or two physical activity limitations per month (β = −0.14) None of these three coefficients was statistically significant.

**Table 4:** Regression coefficients representing preferences of parents and adolescents for attributes of asthma control

Attribute	Parents (*n* = 52)				Adolescents (*n* = 44)			
	Beta	Lower CI	Upper CI	*P*-value	Beta	Lower CI	Upper CI	*P*-value
Night Time Symptoms								
None	2.09	1.64	2.55	<0.00001	1.91	1.40	2.42	<0.00001
3 days per week	−0.18	−0.61	0.24	0.40	−0.25	−0.73	0.23	0.32
5 days per week	−0.90	−1.33	−0.46	<0.00001	−1.15	−1.62	−0.69	<0.00001
Wheezing or tightening of chest								
None	1.88	1.42	2.34	<0.00001	1.43	0.92	1.94	<0.00001
Manageable	0.68	0.23	1.12	0.00290	0.55	0.05	1.04	0.03000
Bothersome	−0.87	−1.29	−0.45	<0.00001	−1.15	−1.61	−0.70	<0.00001
Changing Medication								
None	1.02	0.58	1.47	<0.00001	1.26	0.76	1.77	<0.00001
More doses/add medication	0.11	−0.33	0.56	0.62	−0.03	−0.52	0.47	0.92
Add oral steroids for 5 days	−0.20	−0.66	0.26	0.39	0.02	−0.49	0.53	0.94
Emergency room visits								
None	1.82	1.37	2.28	<0.00001	1.90	1.39	2.41	<0.00001
4 per year	−1.14	−1.55	−0.73	<0.00001	−1.17	−1.62	−0.71	<0.00001
10 per year	−2.15	−2.58	−1.73	<0.00001	−1.83	−2.28	−1.39	<0.00001
Physical activity limitations								
None	1.31	0.85	1.77	<0.00001	2.17	1.66	2.68	<0.00001
2 per month	−0.14	−0.60	0.32	0.54	−0.29	−0.78	0.21	0.26
10 per month	−1.14	--	--	--	−1.97	--	--	--

Conditional logit regression was used to analyse the choice data. The coefficients for all attribute levels were estimated relative to the reference level of 10 physical activity limitations per month. The estimated coefficients represent respondents’ average preference weights for a given attribute level relative to the reference level. R^2^(0) Goodness of fit for model with no intercept 0.2591 for parent model and 0.2804 for adolescent model

**Fig. 2:**
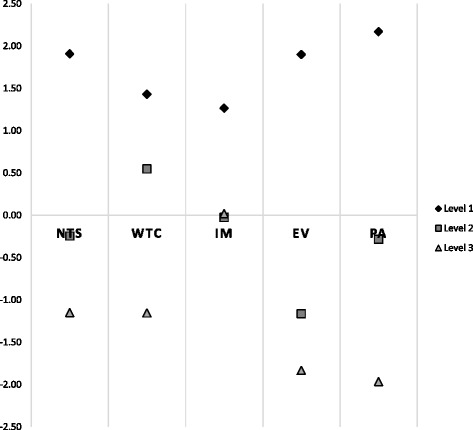
Adolescent preferences for asthma control. The figure indicates the strength of preference of adolescents for each of the levels of each asthma control parameter, with positive values indicating more favoured options. Abbreviations: EV, Emergency room visits; IM, Changing medication; NTS, Night-time symptoms; PA, Physical activity limitations; WTC, Wheezing or tightening of chest

Adolescents’ preferences for asthma control attributes are presented in Table 4 and Fig. 2. While the strength of their preference for averting night-time symptoms was similar to parents, adolescents demonstrated the strongest positive preference for the absence of physical activity limitations (β = 2.17, *p* < 0.00001), which was greater than their preference for the absence of night-time symptoms, the absence of wheezing or chest tightening, no changes to medications, and no emergency room visits. Adolescents’ positive preference for averting emergency room visits (β = 1.90, *p* < 0.00001) was similar to parents (β = 1.82, *p* < 0.00001). Adolescents’ strongest negative preference was for ten physical activity limitations per month (β = −1.97) closely followed by ten emergency room visits per year (β = −1.83, *p* < 0.00001) which was less preferred than frequent night-time symptoms, bothersome wheezing or chest tightening or the need to add oral steroid medications. The wide spread between upper and lower levels for physical activity limitations and for emergency room visits suggests that these two attributes were most important to adolescents, given the options available. Their preferences for levels of night-time symptoms and wheezing or chest tightening were similar. Adolescents had a stronger preference for no changes to medications compared to parents but appeared more indifferent than parents with regard to adding more doses or another medication (β = −0.03) and adding an oral corticosteroid (β = 0.02).

## Discussion

Not only do parents and adolescents have a good understanding of the factors that contribute to loss of asthma control, they have unique preferences with regard to which asthma control parameters are most important. Parents of young children with asthma were considered an important respondent group because asthma frequently presents at a very young age and can be severe, and parents are responsible for ensuring their children receive adequate treatment. While their asthma may be less frequent or severe, adolescents may experience difficulty attaining asthma control as they express a growing independence from their parents.

Whereas parents placed the strongest positive preference on the absence of night-time symptoms, adolescents felt that the absence of physical activity limitations was most important. These findings may reflect parents’ beliefs that frequent night-time symptoms may be more directly related to poor asthma control than other attributes or may in part reflect a desire to avert night-time awakenings to improve their own sleep. It may also be possible that parents did not express a stronger preference to avert physical activity limitations because they are often not present when their children engage in organized physical activity and therefore are not as aware of the impact of this attribute on asthma control. Adolescents’ strong preference to avert physical activity limitations may be related to a desire to maintain strong social relationships with peers and avoid stigmatization. Ten emergency room visits per month was least preferred by parents, and while adolescents were highly averse to frequent emergency room visits, they were most averse to ten physical activity limitations per month. Both groups placed least emphasis on changes in medications as an indicator of poor asthma control.

The parameters of asthma control traditionally featured in pediatric clinical guidelines include the frequency of day-time and night-time symptoms, school absences, use of short-acting beta-agonists, and physical activity limitations [1, 2, 17]. In addition, having one or more severe exacerbation in the last year or poor pulmonary function (defined as forced expiratory volume in 1 second less than 60 % of predicted) are recognized as modifiable risk factors for poor outcomes. The most recent EPR-3 guidelines attempt to clearly distinguish between *impairment*, (i.e. the frequency and intensity of day-time and night-time symptoms, use of short-acting beta agonists for symptom relief, lung function, activity limitations and school absences), and *risk* of acute exacerbation, disease progression, and drug-related adverse events [1].

The distinction between impairment and risk of exacerbation is useful and the inclusion of multiple parameters in each domain underscores the many aspects of disease expression. However, the guidelines provide no indication of the relative importance of the listed asthma control parameters. Common assessment tools such as the Asthma Therapy Assessment Questionnaire [19] and the Asthma Control Test [20] assume that each impairment item listed is equally important and they assign equal weight to them in scoring. No previous study has investigated the relative importance of asthma control parameters. The present study demonstrated that not only do parents and adolescents place different weight on individual asthma control parameters, their preferences differ from each other, with parents’ greatest concern being frequent emergency room visits, and adolescents, frequent physical activity limitations. A study in different samples of parents and adolescents with asthma using a choice experiment with an alternative design aimed to determine whether preferences varied within groups [21]. That study detected two clear classes of respondents within each respondent group demonstrating that parents and adolescents with asthma are not homogeneous with regard to their preferences [21].

Both parents and adolescents displayed the most indifference to changes in medications as indicators of asthma control. Parents had a small positive preference for additional doses or new medications and a slightly negative preference for the need to add an oral corticosteroid. In contrast, the preferences of adolescents for adding doses, new medication or an oral corticosteroid were close to zero, reflecting indifference relative to frequent activity limitations. This perception of changes in medications may be quite different than the views of clinical practitioners who perceive the need for an oral corticosteroid as an important indicator of acute loss of asthma control. The most recent Canadian guidelines recommend that the use of daily controller therapy be promptly reassessed in any patient requiring an oral corticosteroid and that these patients be referred to a specialist in the case of frequent courses of oral corticosteroids [3].

The most recent updates to asthma clinical practice guidelines ask practitioners to consider patient preferences as part of shared clinical decision-making, but this is limited to use of devices such as peak flow meters, medication selection and administration, and other barriers to effective management, such as the cost of medications. Patient preferences with regard to actual asthma control parameters are not considered, even though patients’ and parents’ behaviours, particularly with regard to medication adherence, are strongly influenced by indicators of impairment, such as activity limitations, and indicators of exacerbation, such as emergency room visits [22–24]. The current Grading of Recommendations Assessment, Development and Evaluation (GRADE) guidelines explicitly recommend the examination of patients’ values and preferences in the construction of clinical practice guidelines [25]. The GRADE group recognizes that patients’ preferences, particularly around desired outcomes, adverse effects and trade-offs between them, are essential for promulgating recommendations that are clinically meaningful [25].

For asthma education and management programs to be effective, parents and adolescents must be willing partners, and their views and preferences must therefore be considered in the design of asthma management plans. Adolescents represent a particularly vulnerable group. These individuals possess a growing sense of identity and need for independence while they cope with accepting responsibility for managing their asthma [26]. Their desire to conform to peer expectations and a sense of invulnerability may make them unwilling to use inhaler devices or perceive the need to manage a chronic condition. This research demonstrates the importance of studying the preferences of adolescents as well as the psychosocial factors that influence behaviour [24, 26, 27].

The study included several strengths and limitations. The comparison was not between two patient groups; rather it contrasted parent preferences with adolescent patient preferences. As informal caregivers, parents may have unique preferences that may be unrelated to the age of their child. While it would not have been feasible to directly assess the preferences of children with asthma aged 2 to 12 years with the BWS instrument, it would be of interest to survey parents of adolescents with asthma and compare their preferences with the other two groups. The samples of parents and adolescents were not generated randomly however purposive sampling from the community and hospital-based asthma clinics resulted in samples representing a wide range of socioeconomic, medical history and asthma severity characteristics. The sample sizes for parents and adolescents were not large; larger samples would have increased the statistical efficiency (i.e. reduced the standard errors of the parameters). However, a high level of statistical efficiency was achieved by ensuring that the instrument displayed orthogonality (i.e. the difference in the levels of each attribute varied independently over choice sets), and balance, to confirm that all attribute levels appeared with equal frequency [15]. In addition, measurement error was minimized by thorough pilot testing and feasibility assessment, and by limiting the number of choice tasks to nine. The samples were drawn from urban and suburban communities within and around greater Toronto which may restrict generalizability to areas where asthma management practices are different. In the present study, drug benefits were available to 82 % of parents and 78 % of adolescents. Therefore, for most respondents a lack of medication insurance was not a factor in medication adherence. This may not be the case in other jurisdictions.

## Conclusions

This study revealed that parents of young children with asthma and adolescents with asthma possess unique preferences with regard to averting night-time symptoms, wheezing and chest tightening, changing medication, emergency room visits and physical activity limitations. Parents had stronger preferences for averting night-time symptoms while avoiding frequent physical activity limitation was most important to adolescents. Measuring and understanding the preferences of key stakeholder groups such as parents and adolescents is critical to ensuring the effectiveness of asthma management plans. Incorporating consideration of parent and patient preferences in the design of practice guidelines may also improve their uptake and value. Future research should measure preferences of additional groups, including parents of adolescents and health care providers.

## Availability of data and materials

This clinical data set is not publically available to preserve research subject privacy and confidentiality.

## Additional file

Additional file 1:
**Illustration of choice options for each task and hypothetical best and worst choices for three respondents.** (DOCX 19 kb)

## Abbreviations

AL: Anti-leukotriene
ASC: Asthma Society of Canada
BD: Bronchodilator
BWS: Best worst scaling
CI: Confidence interval
EPR: Expert panel report
EV: Emergency room visits
GINA: Global Initiative for Asthma
GRADE: Grading of recommendations, assessment, development and evaluation
ICS: Inhaled corticosteroid
IM: Changing medication
NTS: Night-time symptoms
PA: Physical activity limitations
SD: Standard deviation
Sick Kids: The Hospital for Sick Children, Toronto, Canada
WTC: Wheezing or tightening of chest
