# Supplementary material for: Parents and adolescents preferences for asthma control: a best-worst scaling choice experiment using an orthogonal main effects design
Source: BMC Pulm Med. 2015 Nov 17;15:146. doi: 10.1186/s12890-015-0141-9 (PMC4650923; doi:10.1186/s12890-015-0141-9)
Supplement: Additional file 1: — Illustration of choice options for each task and hypothetical best and worst choices for three respondents. (DOCX 19 kb) [file 12890_2015_141_MOESM1_ESM.docx]

Technical Appendix. Illustration of choice options for each task and hypothetical best and worst choices for three respondents.

| **Subject No.** | **Block** | **Set** | **Best** | **Worst** | **Attribute level values** | | | | |
| --- | --- | --- | --- | --- | --- | --- | --- | --- | --- |
|  |  |  |  |  | **Night-time Symptoms, days per week [NTS]** | **Wheezing or tightening of chest [WTC]** | **Changes in Medication [IM]** | **Emergency room visits per year [EV]** | **Physical activity limitations per month [PA]** |
| P-001 | 1 | 1 | EV | NTS | 5 | bothersome | oral steroids | 0 | 2 |
| P-001 | 1 | 2 | PA | EV | 0 | none | none | 10 | 0 |
| P-001 | 1 | 3 | EV | NTS | 5 | none | add medication | 0 | 0 |
| P-001 | 1 | 4 | EV | PA | 0 | manageable | none | 0 | 2 |
| P-001 | 1 | 5 | WTC | PA | 3 | manageable | add medication | 4 | 10 |
| P-001 | 1 | 6 | PA | EV | 3 | bothersome | add medication | 10 | 0 |
| P-001 | 1 | 7 | WTC | NTS | 5 | none | oral steroids | 4 | 10 |
| P-001 | 1 | 8 | IM | WTC | 3 | bothersome | none | 4 | 2 |
| P-001 | 1 | 9 | NTS | EV | 0 | manageable | oral steroids | 10 | 10 |
| P-002 | 1 | 1 | EV | WTC | 5 | bothersome | oral steroids | 0 | 2 |
| P-002 | 1 | 2 | WTC | EV | 0 | none | none | 10 | 0 |
| P-002 | 1 | 3 | PA | NTS | 5 | none | add medication | 0 | 0 |
| P-002 | 1 | 4 | NTS | PA | 0 | manageable | none | 0 | 2 |
| P-002 | 1 | 5 | IM | PA | 3 | manageable | add medication | 4 | 10 |
| P-002 | 1 | 6 | PA | EV | 3 | bothersome | add medication | 10 | 0 |
| P-002 | 1 | 7 | WTC | PA | 5 | none | oral steroids | 4 | 10 |
| P-002 | 1 | 8 | IM | EV | 3 | bothersome | none | 4 | 2 |
| P-002 | 1 | 9 | NTS | PA | 0 | manageable | oral steroids | 10 | 10 |
| P-003 | 2 | 10 | WTC | EV | 3 | none | oral steroids | 10 | 2 |
| P-003 | 2 | 11 | EV | PA | 3 | none | none | 0 | 10 |
| P-003 | 2 | 12 | NTS | WTC | 0 | bothersome | oral steroids | 4 | 0 |
| P-003 | 2 | 13 | PA | NTS | 5 | manageable | none | 4 | 0 |
| P-003 | 2 | 14 | PA | EV | 5 | manageable | add medication | 10 | 2 |
| P-003 | 2 | 15 | IM | EV | 5 | bothersome | none | 10 | 10 |
| P-003 | 2 | 16 | NTS | EV | 0 | none | add medication | 4 | 2 |
| P-003 | 2 | 17 | EV | PA | 0 | bothersome | add medication | 0 | 10 |
| P-003 | 2 | 18 | EV | NTS | 3 | manageable | oral steroids | 0 | 0 |

Abbreviations: EV, Emergency room visits; IM, Changing medication; NTS, Night-time symptoms; PA, Physical activity limitations; WTC, Wheezing or tightening of chest.
